# The Performance Evolutions and Mechanism Analysis of Ultra-High-Performance Concrete (UHPC) Matrix Containing Varying Contents of Lithium Slag

**DOI:** 10.3390/ma19132770

**Published:** 2026-06-30

**Authors:** Qiuyu Liu, Yue Li, Guosheng Zhang, Fengkai Ge, Shijun Ding, Jia Sun, Tiantian Chen, Hui Lin

**Affiliations:** 1 College of Architecture and Civil Engineering, Beijing University of Technology, Beijing 100124, China; 2 State Grid Electric Power Engineering Research Institute Co., Ltd., Beijing 100124, China; 3 Jianyan Building Materials Co., Ltd., China Academy of Building Research, Beijing 100013, China

**Keywords:** ultra-high-performance concrete, lithium slag, mechanical properties, microstructure, mechanistic analysis

## Abstract

With the continuous expansion of the lithium industry, lithium slag (LS) has been generated in large quantities, and its potential reuse in cement-based materials has become increasingly important. In this work, LS was introduced into ultra-high-performance concrete (UHPC) as a partial substitute for cement to explore its applicability in low-cement UHPC systems. The fresh properties of UHPC, including flowability and setting behavior, were measured, and its mechanical performance was evaluated through compressive and flexural strength tests. In addition, early-age autogenous shrinkage was monitored to clarify the effect of LS on dimensional stability. To further reveal the mechanisms associated with the macroscopic performance changes, isothermal conduction calorimetry, backscattered electron microscopy (BSE), X-ray diffraction (XRD), thermogravimetric analysis (TGA), and mercury intrusion porosimetry (MIP) were adopted. These techniques were used to characterize the hydration behavior, phase composition, hydration product evolution, and pore-structure characteristics of UHPC containing different LS contents. Results demonstrate that LS incorporation effectively reduces autogenous shrinkage and accelerates setting. An optimal LS content enhances long-term strength development; however, LS incorporation compromises early-age compressive strength and flowability. Calorimetric, thermogravimetric, and BSE analyses collectively reveal that LS retards early hydration heat release and delays initial strength gain, attributable to its dilution effect, but exhibits latent pozzolanic reactivity, consuming Ca(OH)_2_ and promoting secondary C–S–H formation, thereby increasing the 28-day degree of cement hydration. Pore-structure analysis further confirms that an appropriate LS content significantly reduces total porosity, average pore diameter, and the volume fraction of pores with PD > 50 nm, leading to a more refined and compact microstructure. Integrated macroscopic and microscopic evidence identifies 20 wt.% LS as the optimal replacement level: relative to the reference mixture LS0, the 28-day compressive and flexural strengths of LS20 are increased by 10.86% and 27.93%, while flowability decreases by 13.97%, initial setting time shortens by 10.54%, and autogenous shrinkage is reduced by 57.41%. The results provide a scientific basis for the resource utilization of lithium slag in UHPC and contribute to the development of cement-reduced UHPC mixtures with improved mechanical and microstructural characteristics.

## 1. Introduction

Ultra-high-performance concrete (UHPC) is an advanced cement-based composite material fabricated using high-volume cementitious materials, fine aggregates, high-efficiency water reducers, and fibrous materials under an extremely low water-to-cement ratio [1]. In comparison to conventional concrete, UHPC exhibits exceptional compressive and tensile strengths, prominent tensile strain-hardening behavior, and ultra-low permeability [2,3,4]. Because of these advantages in strength and durability, UHPC has been increasingly used or considered in bridge engineering, prefabricated structures, architectural components, and other engineering applications [5,6,7].

In conventional UHPC, the combined use of a high cement dosage and a very low water-to-cement ratio usually limits cement hydration, with the hydration degree generally remaining at only 30–40% [8]. Consequently, a large fraction of unhydrated clinker particles act not as reactive binders but as inert micro-aggregates, contributing solely to physical packing. This low utilization efficiency entails substantial embodied energy and CO_2_ emissions per unit strength gain, representing both an environmental burden and a material inefficiency. Moreover, the excessive clinker content intensifies early-age autogenous shrinkage due to rapid internal relative humidity drop and self-desiccation; the resulting microcracks compromise structural integrity, accelerate chloride ingress, and undermine long-term durability. These limitations critically hinder the practical deployment of conventional UHPC in monolithic applications. Accordingly, reducing the cement dosage in UHPC through partial substitution with supplementary cementitious materials or inert fillers is of considerable environmental and economic significance.

To advance the sustainable development of UHPC, numerous researchers have initiated investigations into the fabrication of low-cement UHPC. Recently, various industrial by-products, such as ground granulated blast-furnace slag (GGBS) [9], steel slag (SS) [10], fly ash (FA) [11], and limestone powder (LP) [12], have been rigorously investigated as supplementary cementitious materials (SCMs) or inert fillers for partial clinker substitution in UHPC systems. Moula et al. reported that when GGBS replaced 30% of the cement, the density of the concrete was improved, its flowability was enhanced, and the setting speed was accelerated [13]. Yalçınkaya and Opurolu demonstrated that incorporating GGBS markedly suppressed the hydration heat evolution of UHPC, delayed early-age compressive strength development, and had little effect on its 28 d strength [14]. In the study by Zhang et al., SS was used to partially replace cement in UHPC. When the SS content reached 30 wt.%, the 1-day and 28-day strengths declined by 45.5% and 5.8%, while autogenous shrinkage was lowered by 18.21%. The SS increased the porosity and coarsened the pore structure of UHPC, which had an adverse effect on the mechanical properties and durability of UHPC [15]. Abdellatief et al. examined the combined use of FA, GGBS, and metakaolin (MK) as supplementary cementitious materials in UHPC. Their results indicated that the cement proportion in the binder system could be reduced to 35–65 wt.% without compromising mechanical performance [16]. Yu et al. employed limestone powder (LP) as a cement replacement in UHPC and reported that a 30 wt.% LP dosage markedly improved workability while maintaining mechanical strength and increasing the cement hydration degree [17]. With the increasing utilization of industrial by-products in cementitious systems, these materials have gradually been transformed from industrial waste to valuable resources with practical application value [18]. However, industrial by-products that can be applied to the cement and concrete industry sometimes face supply shortages, and the incorporation of some by-products may adversely affect the macroscopic properties of UHPC. Accordingly, identifying additional alternative binders is necessary to broaden and improve the current cementitious material system.

Some specially treated solid wastes have also been used as alternative materials to cement for the preparation of UHPC. Biomass-derived ashes have also been explored as cement substitutes in UHPC systems, with typical examples including rice husk ash [19], bagasse ash [20], and palm leaf ash [21]. Biomass ash, similar to industrial wastes such as FA, SF, and GGBS, may contribute to comparable or improved performance in cementitious systems. However, the properties of biomass ash are highly dependent on combustion conditions and the presence of soil-derived impurities [22]. In addition, mechanical grinding is generally required to fully activate its pozzolanic reactivity [23], which may restrict its large-scale use in UHPC.

Construction waste has also been considered as an alternative raw material for UHPC production. In the study by Wang et al., construction waste was used to partially replace the binder and aggregate fractions in UHPC. When the replacement levels reached 50% for cement and 19% for fine aggregates, the resulting UHPC maintained a compressive strength similar to the reference mixture, accompanied by a denser microstructure, lower early hydration heat, and reduced autogenous shrinkage [24]. In the work of Yuan et al., recycled brick powder (RBP) was added to UHPC at varying contents, and its influence on mechanical properties was evaluated. The addition of RBP was found to be unfavorable to compressive strength development [25]. Lu et al. reported that incorporating crushed glass and glass powder reduced the mechanical properties of UHPC, but markedly decreased its density and improved its thermal insulation performance [26]. However, not all construction waste is suitable as an auxiliary cementitious material for UHPC preparation. For instance, the high crushing value and water absorption rate of waste clay brick aggregates severely restrict their large-scale application in concrete [27]. Moreover, most construction waste needs to be sorted and ground before use, and this process itself is accompanied by additional resource and energy consumption.

Lithium slag (LS) is a solid industrial by-product generated during the extraction of lithium carbonate and lithium hydroxide from lithium ores. The production of 1 ton of lithium carbonate is typically accompanied by the generation of about 9 tons of lithium slag [28]. In China, LS stockpiles have accumulated to about 1.2 billion tons. As the global lithium industry continues to expand, lithium extraction is predicted to peak by 2060. Consequently, substantial amounts of LS are likely to be generated and stockpiled for decades [29]. The massive stockpiling of LS may pose potential risks to water and soil environments while also causing considerable resource waste. Therefore, the effective utilization and disposal of continuously increasing LS have become critical issues requiring urgent attention [30]. LS is mainly composed of amorphous SiO_2_ and Al_2_O_3_, with a small amount of CaO and CaCO_3_. Owing to this composition and its pozzolanic activity, LS can be used as a supplementary cementitious material in cement-based systems [31]. Recent studies have further confirmed that LS can participate in secondary hydration reactions, consume Ca(OH)_2_, promote the formation of additional C–S–H/C–A–S–H gels, and refine the pore structure of cement-based materials [32,33]. Existing research also shows that LS has considerable potential as a mineral admixture for improving the mechanical properties, microstructure, and durability-related performance of ordinary concrete at appropriate replacement levels [34,35,36].

However, most existing studies have focused on ordinary cement paste, mortar, and conventional concrete systems. In contrast, research on the use of LS as a high-volume cement substitute in UHPC remains limited, especially regarding its coupled effects on fresh properties, early-age autogenous shrinkage, hydration kinetics, pore-structure evolution, and strength development. Meanwhile, the development of low-cement and low-carbon UHPC has received increasing attention, with recent studies emphasizing the use of industrial by-products and supplementary cementitious materials to reduce cement consumption while maintaining dense microstructures and high mechanical performance [37,38]. Therefore, as a supplementary cementitious material with both resource-utilization and economic advantages, LS shows promising potential for application in cement-reduced UHPC systems.

In this study, six UHPC mixtures containing 0–50 wt.% LS as a cement substitute were proportioned according to the modified Andreasen–Andersen (MAA) packing model. The effects of LS on flowability, setting time, autogenous shrinkage, and mechanical properties were systematically evaluated. In addition, XRD, TGA, BSE, and MIP analyses were conducted to clarify how LS content affects hydration products, cement hydration degree, and pore-structure evolution.

## 2. Materials and Methods

### 2.1. Raw Materials

**Figure 1:**
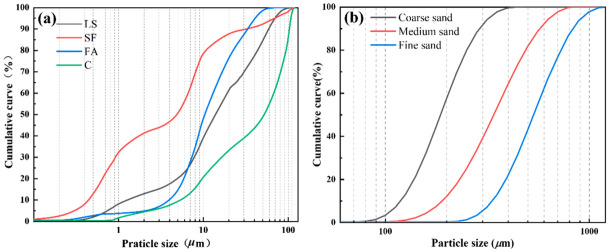
Particle size distributions of (**a**) cementitious materials and (**b**) quartz sand.

In this study, P·II 52.5 Portland cement (C), supplied by Anhui Conch Co., Ltd. (Wuhu, China), served as the primary binder in all UHPC mixtures. The physical properties of cement and the mechanical properties of standard cement mortar are summarized in Table 1. The supplementary binders used in this study consisted of silica fume (SF), grade II fly ash (FA), and lithium slag (LS), supplied by Elkem Co., Ltd. (Oslo, Norway), Platinum Refractory Materials Co., Ltd. (Tangshan, China), and Tangshan Xinfeng Lithium Industry Co., Ltd. (Tangshan, China), respectively. Table 2 summarizes the chemical compositions of the four binders, while Figure 1a compares their particle size distributions. According to Figure 1a, the fineness of LS is slightly higher than that of cement, which can effectively increase the filling density of concrete. Three gradations of quartz sand were employed as fine aggregates: fine sand (0.079–0.56 mm), medium sand (0.11–0.89 mm), and coarse sand (0.22–1.42 mm); their respective grading curves are provided in Figure 1b. The reinforcing fibers were copper-coated short straight steel fibers (diameter of 0.2 mm, length of 14 mm, ultimate tensile strength of 2800 MPa, density of 7800 kg/m^3^, elastic modulus of 200 GPa). To ensure the required workability, a high-performance polycarboxylate-based superplasticizer (water reduction capacity ≥40%) was used.

### 2.2. Mix Design of UHPC

In UHPC mix design, maximizing particle packing density is essential to achieving superior macroscopic performance, and this objective hinges on the rational gradation of all constituent materials across multiple size scales. To achieve a more accurate description of dense particle packing, Funk and Dinger modified the original Andreasen–Andersen equation, from which the MAA model was derived [39]. Owing to its theoretical rigor and experimental verifiability, the MAA model has been widely adopted as a quantitative framework for rational UHPC mixture proportioning [17,40]. In this study, the MAA model was adopted to optimize the basic UHPC mixture proportions by determining the particle size distribution of all granular constituents required for dense packing, as expressed in Equation (1):

(1) $$P(D)=\frac{D^{q}-D_{\min}^{q}}{D_{\max}^{q}-D_{\min}^{q}}$$

where *P*(*D*) denotes the cumulative volume fraction of solid particles with diameters less than *D*; *D* represents the particle diameter (μm); *D*_max_ and *D*_min_ denote the maximum and minimum particle diameters in the system, respectively; and *q* denotes the distribution modulus, which was set as 0.21 in this work.

**Figure 2:**
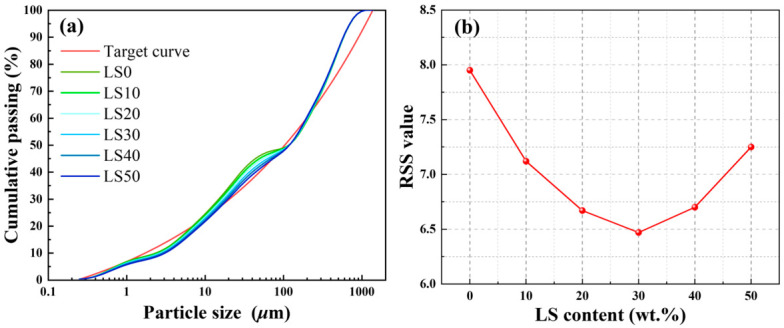
(**a**) Theoretical optimized particle size distribution curves and (**b**) RSS values of UHPC mixtures with different lithium slag contents.

The theoretically optimized particle size distribution (PSD) curve derived from the MAA model is presented in Figure 2a. In the MAA model, the deviation between the cumulative particle size distribution of the mixture and the target curve is used to evaluate the packing compactness of the particle system. The closer the two curves are, the more fully the inter-particle voids are filled. Taking the MAA model as the objective function, MATLAB R2023a, version 9.14 was employed to model Equation (1). The mixture composition was optimized by LSM, in which the proportion of each component was varied iteratively to minimize the deviation between the actual and target gradation curves. The residual sum of squares (RSS) was used as an index to measure the mismatch between the actual grading curve and the target curve, and its calculation formula is as follows:

(2) $$RSS=\sum_{i=1}^{n}(P_{mix}(D_{i}^{i+1})-P_{tar(}(D_{i}^{i+1}){)}^{2}$$

where *RSS* denotes the residual sum of squares; and *P*_mix_ and *P*_tra_ denote the target gradation curve and the actual cumulative curve, respectively.

Figure 2b presents the *RSS* values for UHPC mixtures of varying LS contents. As illustrated, all LS-containing mixtures yield lower RSS values relative to the reference mixture LS0, demonstrating enhanced alignment between their experimentally measured cumulative particle size distributions and the MAA-derived target curve. The complete mixture proportions are tabulated in Table 3, where the mass-based replacement ratio of cement by LS is the sole variable. Specimens are labeled using the nomenclature “LS + X”, with X representing the LS replacement percentage by mass (e.g., LS10 denotes 10 wt.% LS content).

### 2.3. Preparation and Curing of Specimens

First, cementitious materials and quartz sand were weighed according to the designed mixture proportions and introduced into a planetary mortar mixer. Dry mixing was performed for 90 s to achieve homogeneous dispersion of powders and aggregates. After dry mixing, water premixed with the polycarboxylate-based superplasticizer was added stepwise to the mixture, and wet mixing continued for 300 s to ensure complete wetting and uniform coating of aggregate surfaces with cementitious paste. Thereafter, steel fibers were gradually introduced through a 2 mm aperture sieve while mixing continued for an additional 180 s at low speed to guarantee homogeneous fiber distribution without balling or segregation. The fresh mortar was then cast into three-cavity prismatic molds (40 mm × 40 mm × 160 mm) and consolidated on a high-frequency vibration table for 60 s to eliminate entrapped air. Immediately after molding, the specimens were covered with cling film to prevent water evaporation. After standing at room temperature for 24 h, the specimens were demolded and placed in a standard curing box at 20 ± 2 °C and a relative humidity of not less than 95% for curing. The specimens were taken out at ages of 3 days and 28 days for mechanical performance testing.

### 2.4. Experimental Methods

#### 2.4.1. Fresh-Mixed Properties

The mini slump flow of UHPC was determined using a metal truncated cone mold (top diameter: 70 mm, bottom diameter: 100 mm, height: 60 mm) and a jumping table, following the procedures specified in the Chinese standard GB/T 2419-2005. The fresh mortar was filled into the mold in two layers of equal height, and each layer was tamped 15 times with a standard rod to improve compactness and remove entrapped air. Immediately after filling, the mold was lifted vertically and the jumping table was activated for exactly 25 impacts. Within 30 s of the final impact, the diameters of the spread mortar bed in two orthogonal directions were measured to the nearest 1 mm using a stainless-steel ruler, and their arithmetic mean was recorded as the mini slump flow value, representing the workability of the UHPC slurry.

The setting time of UHPC was evaluated according to JGJ/T 70-2009 [41], using an automatic mortar setting time tester. Testing was conducted under controlled ambient conditions of 20.0 ± 2 °C. The time elapsed from the moment of water addition until the penetration resistance of the fresh mortar reached 0.5 MPa was recorded as the initial setting time. For each mixture, two replicate specimens were tested in parallel; the arithmetic mean of the two valid measurements was reported as the representative setting time. A test was deemed invalid if the absolute difference between the two replicates exceeded 30 min, in which case the test was repeated.

#### 2.4.2. Mechanical Properties

**Figure 3:**
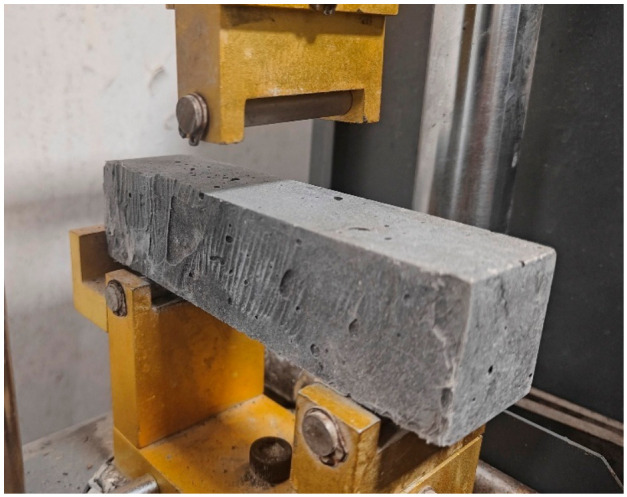
Flexural strength test of UHPC.

The mechanical properties of UHPC were determined following GB/T 17671-2021. For each mixture, 40 mm × 40 mm × 160 mm prism specimens were prepared and tested on a DYE-300S automated universal testing machine (Hebei Huaxin Testing Instrument Co., Ltd., Cangzhou, China). The flexural strength was first obtained by a three-point bending test. During the flexural test, the specimen was simply supported on two lower rollers with a support span of 100 mm, and the load was applied vertically at the midspan through an upper loading roller (Figure 3). The test was conducted under load-control mode at a loading rate of 50 N/s. The testing machine recorded the applied load, and the maximum load at failure was used to calculate the flexural strength. After bending failure, the two broken halves of each prism were collected for compression tests. The compression tests were also conducted under load-control mode at a loading rate of 2400 N/s. The testing machine recorded the applied load during compression, and the peak load at failure was used to calculate the compressive strength. Three prisms were used to calculate the average flexural strength, while six broken halves were used to determine the average compressive strength.

#### 2.4.3. Shrinkage Behavior

Autogenous shrinkage of UHPC was measured using the corrugated tube method. A low-density polyethylene corrugated tube (outer diameter 30.5 mm, effective length 420 mm, wall thickness 0.5 mm) was employed. The tube featured triangular corrugations with a pitch of 6 mm, designed to minimize contact area with the supporting frame and thereby reduce friction-induced constraint. Both ends were sealed with conical rubber stoppers and reinforced with waterproof sealing tape to ensure hermeticity. Electronic dial gauges were used to measure the autogenous shrinkage. Tests were conducted in a temperature and humidity-controlled chamber (20.0 ± 2.0 °C, RH = 60 ± 5%). Autogenous shrinkage strain was calculated from the recorded displacements and reported as microstrain (*μ*ε). The autogenous shrinkage data were immediately recorded at intervals of 30 min for 96 h. The measurement protocol employed an optimized time-zero definition, taking the time of the first measurement of length change after casting following UHPC casting into corrugated tubes rather than the conventional final setting time. This approach reflects more accurate autogenous shrinkage characterization by accounting for bleeding water of fresh UHPC, enabling better detection of early-stage shrinkage behavior [42,43].

#### 2.4.4. Microstructural Characterization

The influence of varying LS contents on the reaction degree and reaction rate of the UHPC matrix was investigated using an ATM AIR isothermal calorimeter (TA Instruments, New Castle, DE, USA). For each mixture, 10 g of the blended cementitious materials was weighed according to the designed proportion, transferred into a plastic container, and then placed in the calorimeter to record heat flow and cumulative heat release. Afterward, deionized water and the polycarboxylate-based superplasticizer were introduced successively, and the paste was mixed until uniform. Thermal monitoring commenced 15 min after water addition and continued uninterrupted for 90 h under isothermal conditions (20.0 ± 0.1 °C). Heat flow (mW/g) and cumulative heat release (J/g) were recorded continuously at 5 s intervals.

The phase composition, microstructure, pore structure, and thermal stability of the 28-day standard-cured UHPC specimens were characterized using XRD, BSE imaging, MIP, and TGA, respectively. Hydration was terminated at 28 days by solvent exchange with anhydrous ethanol for 72 h. For XRD and TGA, the samples were crushed and ground into powder with a particle size less than 0.075 mm. The unground crushed matrix samples were used for BSE analysis. Additionally, UHPC cube samples with an edge length of approximately 10 mm were taken for MIP analysis.

XRD measurements were performed on an X’Pert Pro MPD powder diffractometer (Malvern Panalytical, Almelo, The Netherlands) using Cu-Kα radiation. The scanning was conducted over a 2θ range of 5–80° with a step size of 0.02°. TGA was performed using a Netzsch STA449-F1 simultaneous TG-DSC thermal analyzer (NETZSCH-Gerätebau GmbH, Selb, Germany). Approximately 20 mg of each sample was heated from 30 °C to 1000 °C under a nitrogen atmosphere at a rate of 10 °C/min. BSE observations were conducted using a VEGA3 scanning electron microscope (TESCAN) (Brno, Czech Republic). Before the test, the sample was gold-coated, embedded in epoxy resin, and the hardened epoxy resin was polished thoroughly with sandpaper and diamond abrasive. The accelerating voltage was 15 kV. MIP measurements were performed using an AutoPore IV 9500 mercury porosimeter (Micromeritics Instrument Corp., Norcross, GA, USA). The intrusion pressure ranged from 0.10 to 60,000 psia, with a contact angle of 140°, a surface tension of 0.485 N/m, and a detectable pore size range of 3 nm–400 μm.

## 3. Results and Discussion

### 3.1. Fresh-Mixed Properties

**Figure 4:**
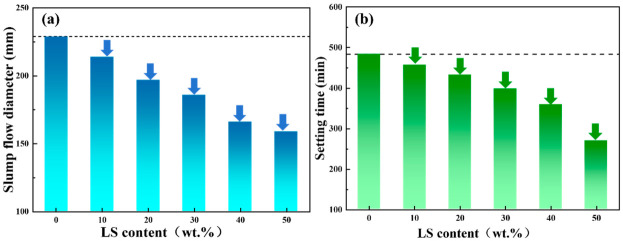
(**a**) Slump flow and (**b**) setting time of UHPC with varying LS contents.

Figure 4a presents the influence of LS content on the mini-slump flow of UHPC mixtures. A progressive reduction in flowability was observed with increasing LS content: from 0 wt.% to 50 wt.%, the mini-slump flow decreases by 30.79%. This decline is primarily attributed to two mechanisms: (i) LS exhibits a high specific surface area and a porous structure [44], leading to increased internal water demand and reduced free water availability for lubrication; and (ii) the angular particle morphology and elevated surface roughness of LS particles intensify interparticle frictional resistance during flow [45].

Figure 4b illustrates the variation in the setting time of UHPC pastes with varying LS replacement levels. The figure shows that substituting cement with LS progressively shortens the setting time, which has a beneficial effect on alleviating the prolonged setting behavior typically caused by the ultra-low water–cement ratio in UHPC [46]. Increasing the LS replacement level from 0 to 50 wt.% shortens the setting time from 484 to 270 min, indicating a 44.21% decrease. This accelerated setting may result from the combined effects of LS reactivity and particle fineness. The reactive components dissolved from LS promote AFt formation during cement hydration [47,48,49], while the finer LS particles absorb more mixing water, thereby reducing free water and shortening the setting time of the paste [45].

### 3.2. Mechanical Properties

**Figure 5:**
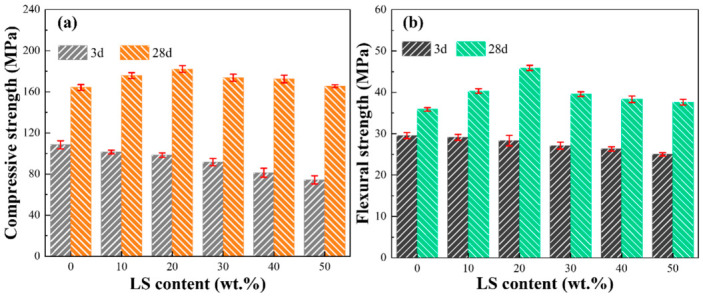
(**a**) Compressive strength values and (**b**) flexural strength values of UHPC with varying LS contents.

The effects of LS replacement level on the compressive and flexural strengths of UHPC at varying curing ages are presented in Figure 5. At 3 d, both strengths exhibit a monotonic decline with increasing LS content: from 0 wt.% to 50 wt.%, compressive strength decreases by 31.35%, and flexural strength decreases by 15.36%. In contrast, the 28-day compressive and flexural strengths of UHPC first increased and then decreased with increasing LS content, peaking at 20 wt.% LS content. The 28-day compressive strength reaches 182.15 MPa (a 10.86% increase over the reference mixture LS0), while the flexural strength attains 45.90 MPa (a 27.93% increase over LS0). The 28-day compressive and flexural strengths of LS50 decreased significantly compared to LS20, but were still slightly higher than those of the reference mixture LS0 by 0.73% and 4.82%. These results demonstrate that the incorporation of LS in UHPC has an adverse effect on the early strength but enhances the later strength. Based on the optimal balance between 28 d strength gain and 3 d strength retention, the recommended LS content is 20 wt.%.

The above-mentioned strength development pattern can be further verified by the microstructural results in Section 3.4, Section 3.5, Section 3.6, Section 3.7 and Section 3.8. The reduction in early-age strength of the LS group can be attributed to the delayed early hydration exothermic reaction, while the increase in 28-day strength of the LS20 group is consistent with the TGA results showing an increase in bound water content, a decrease in Ca(OH)_2_ content, and the denser matrix and finer pore structure observed in the MIP results.

### 3.3. Shrinkage Behavior

**Figure 6:**
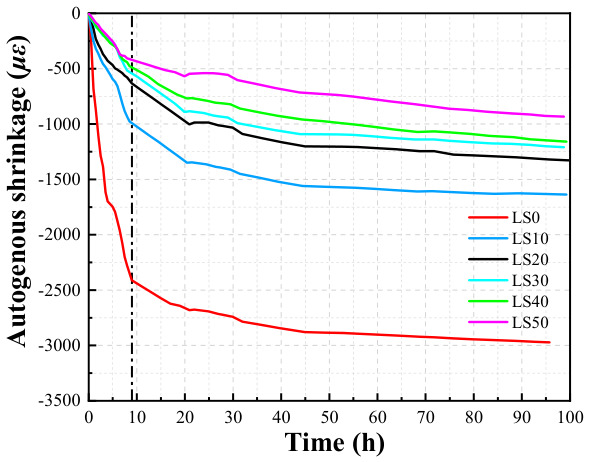
Autogenous shrinkage of UHPC with varying LS contents.

Figure 6 illustrates the influence of LS content on the autogenous shrinkage of UHPC. The autogenous shrinkage of each UHPC mixture develops progressively with time, with comparable curve shapes observed among different LS contents. According to the shrinkage rate, the evolution of autogenous shrinkage can be characterized by two distinct stages: (1) a rapid growth period, during which autogenous shrinkage increases sharply in a short period of time, reaching 50–80% of the 3-day autogenous shrinkage within 7–9 h after the start of the test; (2) a slow growth period, during which the autogenous shrinkage development rate of UHPC gradually decreases, and the curve tends to be flat. LS incorporation markedly reduces the autogenous shrinkage of UHPC, and this reduction becomes more pronounced at higher LS contents. When the LS replacement level increases from 0 to 50 wt.%, the 3-day autogenous shrinkage decreases from 2929.97 × 10^−6^ to 845.33 × 10^−6^, corresponding to a reduction of approximately 71.14%. The reduction in autogenous shrinkage can be further linked to the decrease in cement usage and changes in the hydration process caused by the incorporation of LS. The results of hydration heat and TGA indicate that LS delays the early hydration and reduces the amount of early hydration products, thereby lowering the early shrinkage potential. At the same time, the later pozzolanic reaction and the refinement of pore structure help to maintain or improve the mechanical properties at 28 days.

### 3.4. Reaction Heat Evolution

**Figure 7:**
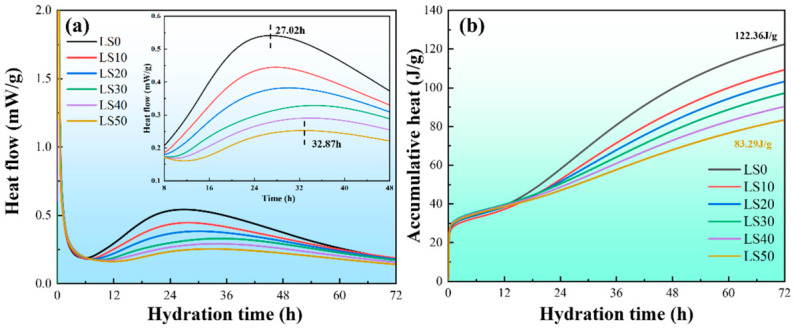
Hydration heat release of UHPC with varying LS contents: (**a**) heat flow; (**b**) accumulative heat.

The influence of LS content on the heat evolution rate of UHPC as measured by isothermal conduction calorimetry is shown in Figure 7a. As the LS content increased from 0 wt.% to 50 wt.%, the magnitude of the main hydration exotherm peak decreases monotonically, from 0.54 mW/g for LS0 to 0.29 mW/g for LS50; while the time-to-peak shifts progressively from 27.02 h to 32.87 h. This suggests that LS exhibits low early-age reactivity in UHPC slurry, thereby slowing the rate of internal relative humidity decline and consequently mitigating early-age autogenous shrinkage.

Figure 7b illustrates the influence of LS content on the accumulative heat release of UHPC pastes over 72 h, as determined by isothermal conduction calorimetry. Compared with LS0, mixtures incorporating LS exhibit lower cumulative heat release, with a further reduction observed at higher LS replacement levels. Increasing the LS replacement level from 0 to 50 wt.% leads to the cumulative heat release of the matrix at 72 h decreasing by 31.93% (from 122.36 J/g to 83.29 J/g). This result indicates that LS incorporation reduces the early reaction degree of UHPC, leading to fewer hydration products and consequently weakening early-age strength, which agrees with the trend shown in Figure 5.

### 3.5. BSE Analysis

In stereological analysis, the phase proportion measured on a representative two-dimensional section can be regarded as an estimate of its three-dimensional volume proportion [50]. Therefore, quantitative BSE image analysis allows the relative content of each phase to be determined from its occupied image area. Because quartz sand particles and pores are not included in the cementitious matrix, their areas were removed from the effective analysis region before calculating the phase fractions. Based on the image analysis method, the expression of the reaction degree of cement clinker at age *t* is as follows [51]:

(3) $$\alpha_{\mathrm{cem}\;}=1-V_{(t)\mathrm{cem}}/V_{(0)\mathrm{cem}}\times 100\%$$

where *V*_(*t*)cem_ denotes the volume fraction (%) of unhydrated cement clinker at curing age *t* calculated, as determined by quantitative BSE image analysis; *V*_(0)cem_ represents the initial volume fraction (%) of cement clinker in the fresh paste prior to hydration. In this study, the expression of *V*_(0)cem_ is as follows:

(4) $$V_{(0)cem}=\frac{\frac{m_{cem}}{\rho_{cem}}}{\frac{m_{cem}}{\rho_{cem}}+\frac{m_{SF}}{\rho_{SF}}+\frac{m_{FA}}{\rho_{FA}}+\frac{m_{LS}}{\rho_{LS}}+\frac{m_{H_{2}O}}{\rho_{H_{2}O}}}\times 100\%$$

where $\rho_{cem}$, $\rho_{SF}$,$\rho_{FA}$, $\rho_{LS}$, and $\rho_{H_{2}O}$ denote the true densities (g/cm^3^) of cement, SF, FA, LS, and water, respectively; $m_{cem}$, $m_{SF}$, $m_{FA}$, $m_{LS}$ and $m_{H_{2}O}$ represent the corresponding masses (g) of cement, SF, FA, LS, and water in a 1000 g composite system.

**Figure 8:**
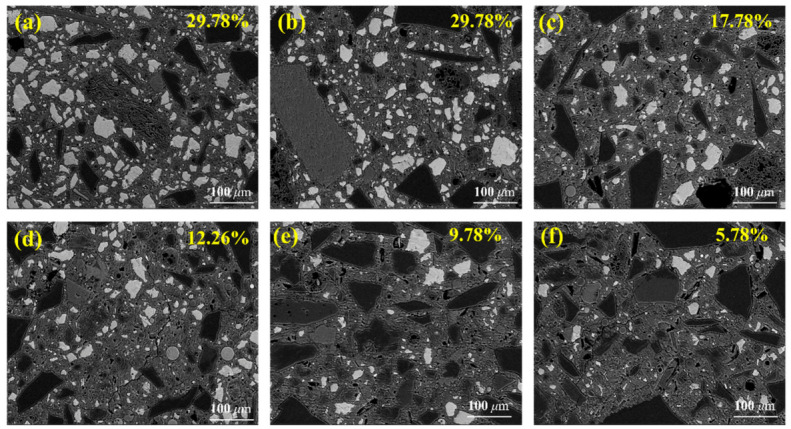
BSE images of UHPC samples at 28 d: (**a**) LS0; (**b**) LS10; (**c**) LS20; (**d**) LS30; (**e**) LS40; (**f**) LS50.

Figure 8 presents the backscattered electron (BSE) images of UHPC samples incorporating LS at varying contents (0–50 wt.%). The white-bright regions correspond to unhydrated cement clinker particles. The percentage value in the upper-right corner of each image denotes the area fraction of unhydrated clinker relative to the total effective analysis area, i.e., the image area excluding quartz sand aggregates and pores. As shown, the area fraction of unhydrated clinker consistently decreases with increasing LS content, suggesting an enhanced dissolution and early hydration of cement clinker in the presence of LS.

**Figure 9:**
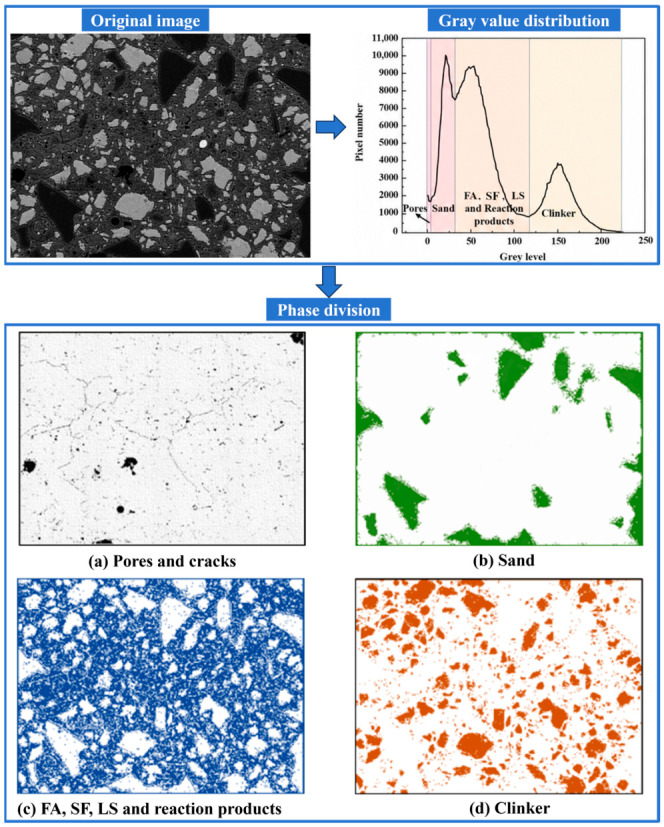
The procedure of threshold segmentation based on BSE images and MATLAB.

Threshold-based image segmentation was performed on backscattered electron (BSE) micrographs using MATLAB^®^ software (R2023a). This procedure enabled the quantitative characterization of phase-specific parameters, including area fraction, morphological descriptors, particle count per unit area, and spatial distribution statistics, thereby facilitating objective discrimination among constituent phases such as unhydrated clinker, hydration products, quartz sand, and pores. Representative segmentation outcomes and the corresponding workflow are presented in Figure 9.

**Figure 10:**
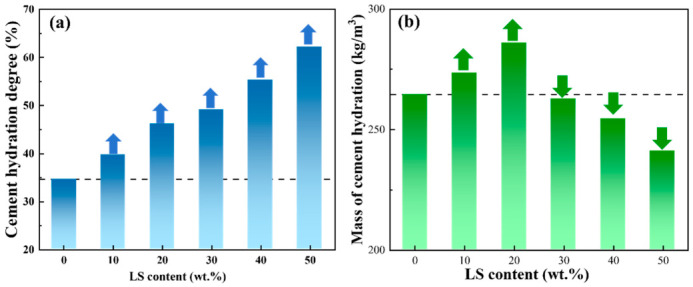
(**a**) Cement hydration degree and (**b**) hydration mass unit volume of UHPC samples at 28 d.

A total of 100 BSE micrographs were acquired per sample, and constituent phases were systematically classified using the aforementioned method. The degree of reaction of cement clinker was computed for each image according to Equation (3), and the arithmetic mean across all images was reported as the representative value for that sample. Figure 10a presents the evolution of clinker reaction degree as a function of LS content. Results indicate that LS incorporation consistently enhances clinker hydration relative to the reference mixture LS0, with the reaction degree increasing monotonically with rising LS content. This trend arises not from direct participation of LS components in cement hydration, but rather from the dilution effect: substituting LS for cement while maintaining constant mixing water increases the effective water-to-cement ratio, thereby promoting more complete clinker dissolution and hydration product formation in UHPC.

Based on the designated mixture proportions, the mass of cement undergoing hydration per cubic meter of UHPC was quantified for each composition; results are summarized in Figure 10b. As shown, the hydrated cement mass exhibits a non-monotonic trend with LS content: it initially rises, peaks at LS20, and subsequently declines. Among all mixtures, LS20 shows the maximum hydrated cement mass, with an 8.15% increase relative to LS0. In contrast, LS50 presents the minimum value, which is 8.86% below that of LS0. This enhancement can be ascribed primarily to two synergistic mechanisms: First, the intrinsic microporosity of LS confers superior water retention capacity [35], which helps retain internal moisture at early ages and maintains sufficient water supply for continued cement hydration. Second, although LS contributes little to early cement hydration, its latent pozzolanic activity becomes evident at later ages. Active Si–Al components from LS consume hydration-derived Ca(OH)_2_ and promote the formation of secondary C–S–H and C–A–H gels, which further densify the matrix [52]. This secondary reaction not only refines the microstructure but also shifts the hydration equilibrium, driving further dissolution of clinker minerals and elevating the overall degree of cement reaction. Beyond 20 wt.% LS, however, the progressive reduction in cement content dominates the response: the absolute mass of cement decreases linearly with LS substitution, while excess LS particles impede interfacial water diffusion, hinder direct cement–water contact, and introduce physical shielding effects that collectively restrict clinker accessibility to water, ultimately suppressing the extent of hydration per unit volume.

### 3.6. XRD Analysis

**Figure 11:**
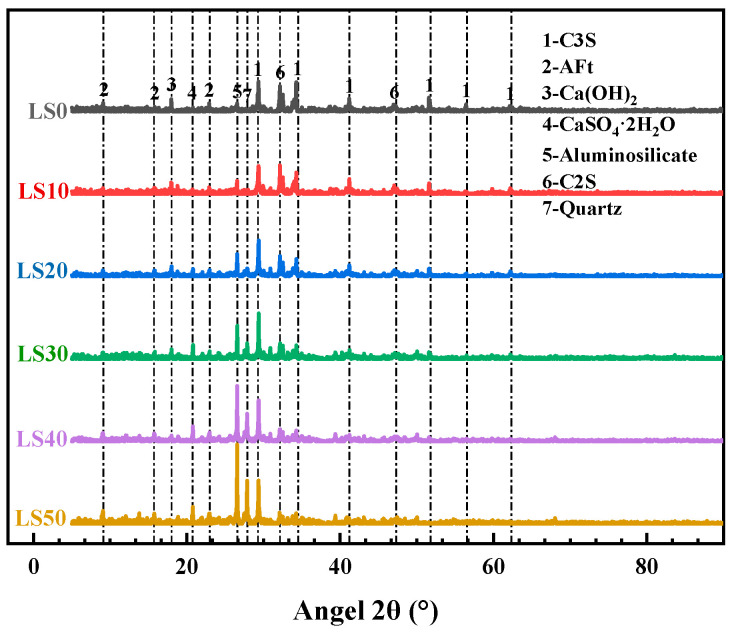
XRD patterns of UHPC samples at 28 d.

Figure 11 presents the XRD patterns of UHPC specimens cured for 28 days, incorporating LS at varying contents (0–50 wt.%). All samples exhibit qualitatively similar phase assemblages, dominated by C_3_S, C_2_S, and Ca(OH)_2_ generated by the hydration of cement clinker, and the reaction product ettringite (AFt). With increasing LS content, the integrated intensities of the characteristic diffraction peaks corresponding to Ca(OH)_2_, C_3_S, and C_2_S progressively diminish. This attenuation is accompanied by a progressive rise in AFt peak intensity, attributable to sulfate ions (SO_4_^2−^) and reactive alumina (Al_2_O_3_) from LS [53,54], which promote AFt formation. Moreover, diffraction signals from gypsum dihydrate (CaSO_4_·2H_2_O), aluminosilicate phases, and crystalline quartz intensify proportionally with LS content, consistent with their identification as major constituent minerals of the lithium slag. To quantitatively assess the influence of LS on the absolute contents of key hydration products, TGA was performed, as detailed in Section 3.7.

### 3.7. TGA

**Figure 12:**
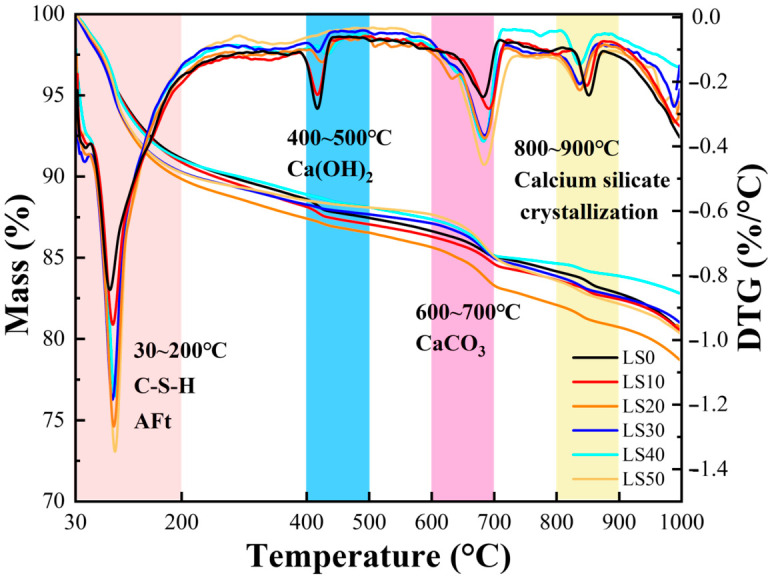
TG/DTG curves of UHPC samples at 28 days.

Figure 12 compares the thermal decomposition behavior of UHPC specimens containing 0–50 wt.% LS after 28 days of curing, as reflected by the TG and DTG curves. The TG/DTG curves reveal four main mass-loss stages for UHPC. The first stage below 200 °C is mainly related to the dehydration of C–S–H gel and partial decomposition of AFt [55]. The second stage, between 400 and 500 °C, corresponds to the dehydroxylation of Ca(OH)_2_ [55]. The mass loss observed at 600–700 °C is assigned to the decomposition of CaCO_3_ [56]. At 800–900 °C, dehydrated calcium silicate hydrates crystallize and transform into more stable calcium silicate phases [57].

The TG curves were analyzed according to Equations (5) and (6) [58] to obtain the content of bound water and Ca(OH)_2_ in each group of samples:

(5) $$W_{b}=\frac{m_{50}-m_{500}}{m_{500}}\cdot 100\%$$

(6) $$CH=\frac{m_{initial}-m_{final}}{m_{final}}\cdot \frac{74}{18}\cdot 100\%$$

where $m_{50}$ and $m_{500}$ denote the sample mass (g) measured at 50 °C and 500 °C, respectively; and $m_{initial}$ and $m_{final}$ represent the sample mass (g) at the onset and completion of Ca(OH)_2_ dehydroxylation, respectively.

**Figure 13:**
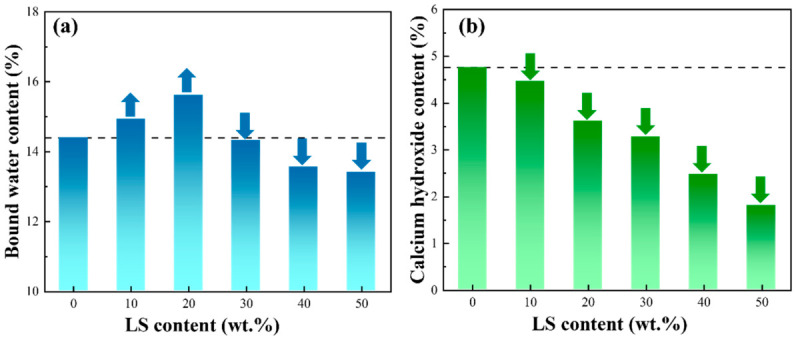
(**a**) Bound water content and (**b**) CH content in UHPC samples with varying LS contents.

Figure 13a presents the change in bound water content as a function of LS dosage. The bound water content rises continuously from LS0 to LS20, reaches a maximum value of 15.61%, and then decreases when more LS is incorporated. The upward trend observed before 20 wt.% LS indicates that moderate LS replacement promotes the generation of hydration products in the UHPC matrix. This improvement is likely related to the denser particle packing and the latent pozzolanic activity of LS, both of which are beneficial for continued hydration. However, once the LS dosage is higher than 20 wt.%, the dilution effect becomes dominant. In this case, the lower cement fraction restricts further hydration product formation, resulting in a reduction in bound water content.

Figure 13b illustrates the Ca(OH)_2_ content in UHPC at varying LS contents. At 28 days of curing, the Ca(OH)_2_ content exhibits a monotonic decline with increasing LS content. BSE reveals that the mass of hydrated cement per unit volume varies by less than ±10% across all groups relative to the control group, indicating that the total amount of Ca(OH)_2_ generated by cement hydration in each group of UHPC samples should be roughly equivalent. However, the Ca(OH)_2_ content in the LS10, LS30, and LS50 groups decreased by 16.95%, 36.44%, and 62.71%, respectively, relative to the reference mixture LS0. This observation further corroborates the pozzolanic reactivity of LS, wherein its active components consume Ca(OH)_2_ to form additional C–S–H gel and other secondary reaction products, thereby reducing the Ca(OH)_2_ content in UHPC. Previous studies have demonstrated that the bonding strength between straight fibers and the matrix increases exponentially with decreasing calcium hydroxide content [59], establishing a key mechanistic basis for the substantial enhancement in flexural strength imparted by LS incorporation. Concurrently, the supplementary C–S–H gel and other secondary reaction products generated from the pozzolanic reaction of LS effectively fill capillary pores, refine the pore structure, enhance matrix densification, and consolidate the solid-phase skeleton, thereby mitigating autogenous shrinkage while contributing to increased compressive strength in UHPC.

### 3.8. Pore-Structure Analysis

**Figure 14:**
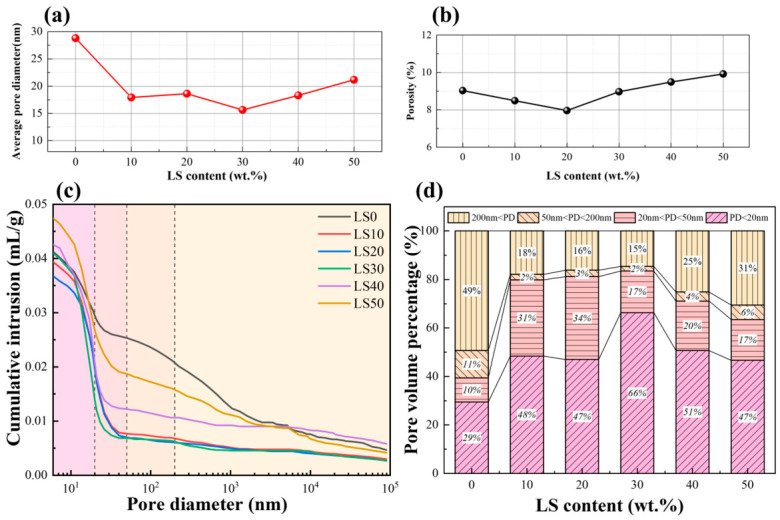
Pore structure of UHPC with varying LS contents at 28 days: (**a**) average pore diameter; (**b**) porosity; (**c**) cumulative pore volume curve; (**d**) pore content.

To elucidate the influence of LS on the microstructural evolution of UHPC, the pore structure of specimens incorporating varying LS contents was characterized by MIP. Cumulative pore volume distributions, porosity, and average pore diameter measured at 28 days are presented in Figure 14. As shown, total porosity exhibits a non-monotonic dependence on LS content: it initially decreases, reaches a minimum at LS20, and subsequently increases. Specifically, LS20 achieves the lowest porosity (7.96%), representing an 11.9% reduction relative to the reference LS0 formulation (9.03%). The LS40 and LS50 exhibit porosities of 9.49% and 9.92%, respectively, exceeding the reference mixture LS0 by 5.1% and 9.8%. The average pore diameter also exhibited a similar pattern. It reached its minimum value (15.61 nm) when the LS content was 20 wt.%, which is approximately 45.8% lower than the reference mixture LS0 (28.80 nm). The average pore diameters of LS40 and LS50 are higher than those of the other groups containing LS, but still lower than the reference mixture LS0.

To quantify the influence of LS content on pore volume within different pore-size ranges, the pores were divided into four diameter intervals: PD < 20 nm, 20 nm ≤ PD < 50 nm, 50 nm ≤ PD < 200 nm, and PD ≥ 200 nm [60]. The pore-size distribution obtained based on this classification is shown in Figure 14d. LS incorporation reduces the volumetric fraction of pores in the 50–200 nm and ≥200 nm ranges while substantially increasing that of pores in the PD < 20 nm and 20–50 nm ranges, thereby shifting the overall pore-size distribution toward finer scales. This microstructural refinement arises from the pozzolanic reaction between active ingredients from LS and Ca(OH)_2_, which generates supplementary C–S–H gel that occludes larger capillary pores [28]. With increasing LS content, the volumetric fraction of pores with PD ≥ 200 nm decreases initially and exhibits a distinct minimum at LS20 (1.29%), and then rises progressively. When the LS content exceeds 20 wt.%, its ability to improve pore structure diminishes, yet the cumulative volume of pores in the 50–200 nm and ≥200 nm ranges in LS50 remains lower than that of the reference mixture LS0. This reduction in larger pores explains why LS50 exhibits slightly superior mechanical properties compared to LS0. As LS20 has the lowest porosity and the lowest fraction of larger pores, it has the best mechanical properties, which is consistent with the mechanical property test results in Figure 5.

## 4. Conclusions

In this work, UHPC mixtures were prepared by partially replacing cement with LS. The influence of LS content on mechanical performance, fresh behavior, hydration characteristics, microstructure, and dimensional stability was systematically examined. From the above analyses, the main conclusions can be drawn:

(1) LS incorporation reduces the flowability of UHPC mixtures while significantly shortening setting times; this effect intensifies with increasing LS content.

(2) Increasing the LS content leads to a monotonic decrease in both 3-day compressive and flexural strength; in contrast, 28-day compressive and flexural strength exhibit an optimum trend, peaking at an intermediate LS content before declining. Consequently, LS incorporation exerts a retarding effect on early-age strength development, whereas an optimal dosage promotes sustained strength gain at later ages.

(3) The incorporation of LS significantly mitigates autogenous shrinkage in UHPC, with the degree of reduction increasing progressively with LS content. Upon increasing LS content from 0 wt.% to 50 wt.%, the 3-day autogenous shrinkage decreases by approximately 71.14%. This effect arises primarily from two synergistic mechanisms: the retardation of early-age hydration kinetics induced by LS dilution, and the supplementary C–S–H gel generated via pozzolanic reaction, which refines the pore structure, enhances matrix densification, and consolidates the solid-phase skeleton, thereby suppressing volumetric contraction.

(4) LS exhibits measurable pozzolanic activity, consuming Ca(OH)_2_ to generate supplementary C–S–H gel, thereby refining the pore structure and reducing overall porosity in UHPC. With increasing LS content, the early-age hydration heat evolution and Ca(OH)_2_ content of UHPC decrease; the hydrated cement mass and bound water content of cement per unit volume first increase and then decrease; the porosity and the proportion of pores with PD > 50 nm first decrease and then increase.

(5) Considering the integrated performance evaluation across all key indicators, a 20 wt.% LS content delivers the optimal overall balance. At this content, bound water content reaches its maximum; total porosity, average pore diameter, and the proportion of pores with PD > 50 nm attain their minima; and 28-day compressive and flexural strengths are significantly enhanced relative to the reference mixture LS0.

## Figures and Tables

**Table 1:** The main physical properties of cement and mechanical properties of standard cement mortar.

Specific Surface Area (m^2^/kg)	Compressive Strength (MPa)		Flexural Strength (MPa)		Setting Time (min)	
358	7 d	28 d	7 d	28 d	Initial setting time	Final setting time
	48.7	57.8	6.6	8.5	165	253

**Table 2:** Chemical compositions of the binders.

Type	Chemical Composition (wt.%)										
	CaO	SiO_2_	Al_2_O_3_	Fe_2_O_3_	SO_3_	MgO	K_2_O	TiO_2_	Na_2_O	P_2_O_5_	Others
C	63.19	20.04	5.27	3.24	2.59	1.59	0.83	0.23	0.17	0.06	2.06
LS	24.17	45.73	16.14	0.71	11.21	0.89	1.49	0.11	3.02	1.22	2.25
FA	7.75	45.05	27.58	7.14	1.72	1.61	2.05	1.41	1.05	0.46	3.50
SF	0.36	94.65	0.25	0.15	0.69	0.47	0.84	-	0.13	0.17	2.29

**Table 3:** Mix proportion of UHPC (kg/m^3^).

Mixture	C	LS	FA	SF	Fine Sand	Medium Sand	Coarse Sand	Water	Steel Fiber	Superplasticizer
LS0	764	0	113	207	317	286	493	195	156	28
LS10	688	76	113	207	317	286	493	195	156	28
LS20	622	152	113	207	317	286	493	195	156	28
LS30	535	229	113	207	317	286	493	195	156	28
LS40	460	304	113	207	317	286	493	195	156	28
LS50	382	382	113	207	317	286	493	195	156	28

## Data Availability

The original contributions presented in this study are included in the article. Further inquiries can be directed to the corresponding authors.
